# *Chiropractic & Manual Therapies*: a critical review of 20 years as an open-access journal

**DOI:** 10.1186/s12998-025-00595-y

**Published:** 2025-08-27

**Authors:** Bruce F. Walker

**Affiliations:** Murdoch University, College of Health and Education, 90 South Street, Murdoch, WA Australia

**Keywords:** Chiropractic, Manual therapies, Journal

## Abstract

**Background:**

This study reviews the 20-year history (2005–2025) of *Chiropractic & Manual Therapies* as an open-access, peer-reviewed journal. Originally launched in 1992 as *COMSIG Review*, the journal has undergone multiple title changes and is currently financially supported by three chiropractic societies. This review critically examines 20 years (2005–2025) as an open-access online journal and makes recommendations for future growth.

**Methods:**

Data on all published articles in the journal *Chiropractic & Manual Therapies* for the period April 11, 2005, until April 10, 2025 were manually entered into SPSS Version 30.0.0.0 between the dates May 7, 2025 and May 16, 2025. The analysis examined article type, publication year, access numbers, citation counts, and Altmetric scores for each published article. Conclusions were then drawn, and recommendations made.

**Results:**

Of the 800 articles published, research articles comprised the majority (59.1%) of publication type, yet systematic reviews demonstrated significantly higher citation rates and online engagement. There has been strong growth in research outputs over the 20 years. Systematic reviews, debate articles and narrative reviews showed notable higher accesses and impact, than other article types. Open access has broadened global reach with 7.49 million accesses over the 20 years examined. The current Impact Factor is 2.3 (2024), the highest for any chiropractic journal worldwide.

**Conclusion:**

Findings highlight the journal’s substantial contribution to the chiropractic and manual therapy literature and offer insights for future editorial direction and impact enhancement. The journal’s unique role as a society-supported publication has enabled exploration of emerging and controversial topics that have attracted high numbers of accesses indicating popularity and reach.

## Background

Initially a print journal, *Chiropractic & Manual Therapies* commenced under the name of COMSIG Review [1] in November 1992. In March 1996 the publication changed its name to *Australasian Chiropractic & Osteopathy *[2] and then on April 11, 2005 the journal again changed its title to just “Chiropractic & Osteopathy [3]. At this juncture the journal went online and fully open-access, managed by the publisher BioMed Central [4]. At the time of going online the journal was at Volume 13. On January 10, 2011 the journal title became *Chiropractic & Manual Therapies* [5].

The journal has always been a “society publication”, initially for the Chiropractic & Osteopathic Musculoskeletal Interest Group (COMSIG) [1] then later for the Chiropractic & Osteopathic College of Australasia (COCA) [6]. COCA was subsumed into a new Society/Association known as Chiropractic Australia [7] in 2016. This society continues to financially support the journal. In 2010 the *European Academy of Chiropractic* (EAC) [8] and in 2012 *The Royal College of Chiropractors* (UK) [9] also generously financially supported the journal and continue to do so. Between 2015 and 2024 the journal was also financially supported by the Chiropractic Knowledge Hub located in Denmark [10].

Upon moving to the online format in 2005 the journal gained PUBMED [11] status. Later in 2017 the journal was listed by MEDLINE [12] and in 2019 gained an Impact Factor with Clarivate [13]. The journal is listed with a host of other Indexing services [14]. The journal’s content is peer-reviewed and governed by an Editorial team, and publications adhere to BioMed Central’s editorial policies [15], which is a member of the Committee on Publication Ethics (COPE) and endorses the International Committee of Medical Journal Editors (ICMJE) Recommendations for the Conduct, Reporting, Editing and Publication of Scholarly Work in Medical Journals. This study examines the 20 years as an open-access online journal and makes recommendations for the future.

## Methods

Data extracted for the journal included: (1) the number of articles published (corrections and one retraction were not included); (2) the article type including editorials, research, case reports, narrative reviews (included scoping reviews), debate, brief/short reports, commentaries, correspondence, databases, hypotheses, methodology, study protocols, systematic reviews and meeting abstracts; (3) year of publication; (4) number of individual article accesses; (5) number of citations (Web of science) [16]; and, (6) Altmetric score [17]. Altmetric is a platform that tracks and analyses online attention received by research outputs, such as journal articles, datasets, and software, across various online sources. It provides a quantitative measure of engagement beyond traditional citations, including mentions on social media, news outlets, and policy documents. An article with a Altmetric score higher than 20 is considered reasonable [18].

The following data were extracted from the journal’s online URL for the period April 11, 2005, until April 10, 2025 and manually entered into SPSS Version 30.0.0.0 between the dates May 7, 2025 and May 16, 2025. Descriptive statistics were generated for the variables above. In addition, some article types were collapsed to form new variables thus:

1. Editorial, Debate, Commentary, Correspondence, meeting abstracts
2. Research
3. Case reports, Narrative reviews (includes scoping reviews), Brief/short report, Hypothesis
4. Methodology, study protocol, database
5. Systematic reviews

Narrative reviews were included in subset 3 as they are more prone to subjectivity and selection bias than systematic reviews [19]. Scoping reviews were initially included as narrative reviews by the publisher so are also seen in subset 3. Descriptive statistics and a plot of the sum for these collapsed categories for each 4-year period was constructed. In addition, frequency, accesses, citations and Altmetric score of the subsets of article types were tabulated.

## Results

Overall, 800 articles were published in the 20-year period. Of these, research articles were the most common article type (59.12%). The sum, frequency (%), mean/median accesses, citations and Altmetric score of article types are shown in Table 1.

**Table 1:** Sum, frequency (%), mean/median accesses, citations and Altmetric score of each article type

Type of article	Number	Percent	Accesses mean, (median)	Citations mean, (median)	Altmetric score mean, (median)
Research	473	59.12	6559 (4851)	6.1 (0)	9.6 (4)
Narrative review/scoping review	68	8.50	21,073 (15,000)	17.3 (4.5)	31.9 (7.5)
Systematic review	67	8.38	11,763 (9496)	10.6 (0)	34.5 (11)
Commentary	49	6.12	11,645 (7322)	5.3 (0)	14.5 (5)
Case report	41	5.12	16,650 (11,000)	4 (0)	3.7 (2)
Study protocol	32	4.00	7025 (5539)	2.7 (0)	5.3 (4)
Debate	24	3.00	18,825 (11,500)	21.1 (3)	25.3 (19.5)
Methodology	15	1.88	5609 (5510)	10.7 (3)	2.7 (2)
Correspondence	12	1.50	2694 (2717)	0.17 (0)	4 (1.5)
Editorial	8	1.00	5924 (3365)	4 (0)	4.5 (2.5)
Brief/Short report	4	0.50	5360 (5059)	3.5 (2.5)	5.25 (0.5)
Hypothesis	3	0.38	9570 (11,000)	2 (0)	11.3 (9)
Database	2	0.25	7785 (7785)	1 (1)	9.5 (9.5)
Meeting abstracts	2	0.25	3578 (3578)	0.5 (0.5)	4.5 (4.5)
Totals	800	100.00	7,490,100	5799	

Total accesses of all 800 articles were 7,490,100, with mean 9362 and median 6018 per article. Total citations were 5799, with mean 7.25, median 0 per article. Altmetric mean was 13.6 and median 4 per article. The sum, frequency, accesses, citations and Altmetric score of article types collapsed into 5 subsets is seen in Table 2.

**Table 2:** Sum, frequency, accesses, citations and Altmetric score of article types collapsed into 5 subsets

Article type		N	%	Number of accesses mean (median)	Number of citations mean (median)	Almetric score mean (median)
Subset 1	Editorial, Debate, Commentary, Correspondence, meeting abstracts	95	11.9	11,676 (6944)	6.15 (0)	14.82 (5)
Subset 2	Research	473	59.1	6559 (4851)	6.12 (0)	9.62 (4)
Subset 3	Case reports, Narrative reviews (includes scoping reviews), Brief/short report, Hypothesis	116	14.5	18,671 (11,000)	11.73 (1.5)	20.47 (4)
Subset 4	Methodology, study protocol, database	49	6.1	6622 (5510)	5.06 (0)	4.65 (3)
Subset 5	Systematic reviews	67	8.4	11,763 (9496)	10.61 (0)	34.51 (11)
	Totals	800	100.0			

**Fig. 1:**
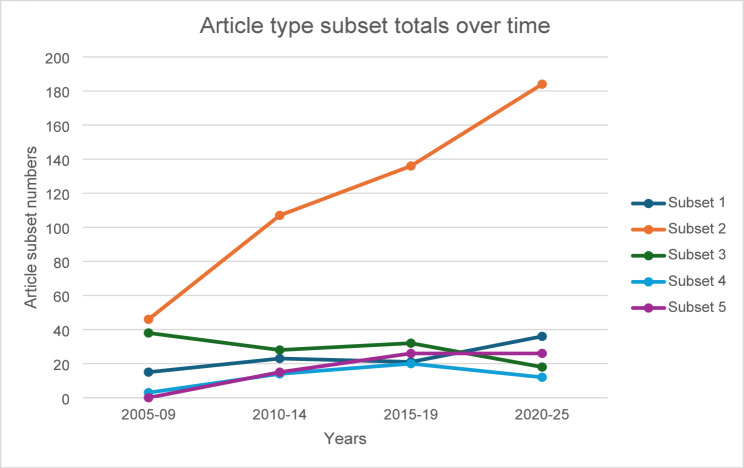
Article type subset totals over time. Subset legend. 1. Editorial, Debate, Commentary, Correspondence, meeting abstracts, 2. Research, 3. Case reports, Narrative reviews (includes scoping reviews), Brief/short report, Hypothesis, 4. Methodology, study protocol, database, 5. Systematic reviews

The plot of the sum for these collapsed categories for each 4-year period is seen in Fig. 1.

## Discussion

*Chiropractic & Manual Therapies* aims (among other things) to publish articles that advance knowledge for the emerging profession of chiropractic and other manual therapy vocations. As a society journal it publishes articles that are not just of interest to the professions but are in the interest of the chiropractic and manual therapy professions and indeed the general public. Achieving a balance between these aims is a challenging one. The journal is managed by an editorial team and has an extensive editorial board selected from a wide range of disciplines [20]. The editorial team must consider choice of peer-reviewers, the scientific processes used, conclusions drawn by authors, the logic and balance proffered in opinion pieces, the rules, regulations and editorial policies of the publisher, and the budget allowed by partner societies to cover some article processing charges. So, with these constraints in mind, the 800 articles published and more than 7 million accesses of those articles in 20 years is impressive.

There has been a strong linear growth in original research articles. Indeed, research articles make up nearly 60% of all articles published. This is a commendable achievement; however, of the 14 article types, research articles rank 10th for accesses, 5th for citations and 6th for Altmetric score. Further, over half of all research articles published were not cited in the Web of Science [16]. Moreover, this was also the case for eight of the 14 article types. So, while research articles are worthy and welcome, the authors may need to, where possible, improve the manuscripts so that there is more likelihood of translation of their results into everyday practice or use in further research. It is acknowledged that some research articles that are not cited may be widely accessed by a clinical audience. It is also recognised that attempts to increase Altmetric scores for the more esoteric research articles is problematic as this requires creating publicity and exposure through social media and other fora.

Systematic reviews constitute Level 1 evidence, so it was pleasing to see that 67 had been published in the past 15 years. Interestingly none were published in the first 5 years online, which may, in part, reflect the paucity of material to unite into a single dataset, and also the emergence of these methods over time. Of note was that the number of systematic reviews published per year did not increase between years 10–20. Given their importance, and the observation that they are highly cited and accessed, the editorial team should consider increasing the commissioning of special and selected systematic reviews. This could be aimed at diagnostic or therapeutic practices within chiropractic and manual therapies where research is equivocal. Initially this could be aimed at those practices still taught at some chiropractic institutions. Also of note was that narrative reviews and scoping reviews were also highly cited and accessed. Scoping reviews are a relatively modern method of combining all information about a topic where there is not a bank of homogeneous studies addressing a similar research question that can be combined in a formal systematic review [21]. It should be noted that narrative reviews/scoping reviews were the second highest cited articles in *Chiropractic & Manual Therapies*. Their application appears suited to many of the under-researched and controversial topics evident in manual therapy professions today. Unfortunately, this subset of article types (subset 3) is the only group that decreased over the 20 years examined. Like systematic reviews the editorial team should consider commissioning scoping reviews about these under-researched topics to advance knowledge and generate debate about such practices. The resulting articles are likely to be widely accessed and cited. Evidence of this likelihood is seen in the journal’s results for “Debate” articles. These articles were the most highly cited of all article types and also had a large number of accesses.

In eight of the 14 article types published, the median score for citations was zero, which meant that over half of each of these 8 article types were not cited in the Web of Science [16]. The editorial team should consider this finding in depth and develop a strategy to improve the metric. This could include developing a list of manuscript elements that enhance citation for use by authors and peer-reviewers. Also, 11 of the 14 article types failed to reach a reasonable Altmetric score of 20 or more. A review of strategies to improve this exposure for articles also needs to be considered by the editorial team.

The placement of the journal as a society publication allows it to explore areas of the manual therapies that may not be possible in a purely scientific publication. In an emerging scientific profession, like chiropractic, this flexibility is of great importance. Funding for the journal to cover Article Processing Charges comes from three professional societies and without this the journal would likely falter. Funding from the societies comes with a commitment to editorial independence. This is a vital characteristic of the running of the journal as it allows it to explore controversial topics without fear or favour.

In 2005 the Editorial Board of the journal chose to publish with an open-access publisher [4]. Articles in open-access journals are available and reusable worldwide free of charge and without restrictions immediately upon publication. Most open access journals charge their authors for publishing articles. In other cases, a hybrid model is used where some articles are subsidised by the journal and others not. This is the model used by *Chiropractic & Manual Therapies*. The content published in open access journals is searchable in the usual databases and search engines. As a rule, contributions in journals that meet this definition are published under a Creative Commons licence granted by the authors [22]. The publishers of these journals are granted only a non-exclusive right of use by the authors [23]. Dissemination of knowledge via open-access journals such as *Chiropractic & Manual Therapies* is enhanced by free access and its availability to all, including those who can least afford it. This is particularly important for clinicians who don’t typically have access to research articles behind pay walls, and lower/middle income countries where resources are limited. This feature is virtuous and should continue to be supported.

This study has limitations, including reliance on data supplied by the publisher within each article. Such data may not be faultless and was not independently validated. Indeed, the use of the publisher’s data concerning the Web of Science citation data may represent an underestimate. However, such data are likely to be indicative and importantly is the database used to establish an Impact Factor. At the time of publication of this article the Impact Factor for this journal was 2.3 (2024), and the 5-year Journal Impact Factor was 2.3 (2024). The journal was ranked in Quartile 1 of the Rehabilitation category of scientific journals. These metrics are the highest for any chiropractic journal worldwide and places the journal in a competitive position with other journals that have a pain and musculoskeletal focus. Future studies of the journal should consider comparing Scopus citations [24] with those from Web of Science as they are likely to be considerably higher. As an example, total Scopus citations for the journal over the 20 years was 11,566 compared to 5799 in the Web of Science. This difference is partly attributable to Scopus including citations in journals that do not have an Impact Factor. In addition, given the emergence of Scoping Reviews, future analysis should provide that these articles form their own category. Article types were arbitrarily collapsed to form new variables, and these subsets may be constituted in other meaningful ways. Data were extracted by only one author but data entry was intermittently checked by another associate. The author also acknowledges that the editorial team may have adopted many or all the recommendations contained herein.

## Conclusion

The stakeholders and team(s) behind the journal *Chiropractic & Manual Therapies* have achieved a great deal in the 20 years since becoming a freely available open access online journal. With continued careful stewardship by the editorial team and editorial board, continued strong independent funding from the journal partners, and support by the publisher, the journal should continue to improve and flourish to greater heights. In this way the journal will continue to bolster the evidence underpinning the professions of chiropractic and manual therapies. The global contribution that *Chiropractic & Manual Therapies* makes should not be under-estimated.

## Data Availability

Data available on request from the author.
